# Interpreting MALDI imaging data for rare types of ampullary cancer using machine learning

**DOI:** 10.1038/s41540-026-00705-3

**Published:** 2026-05-12

**Authors:** Patrick M. Jensen, Jan Lellmann, Christian Sperling, Frieder Meier, Marius Distler, Daniela E. Aust, Herbert Thiele, Pia Hönscheid

**Affiliations:** 1 Department of Applied Mathematics and Computer Science, Technical University of Denmark, Lyngby, Denmark; 2 Institute of Mathematics and Image Computing, University of Lübeck, Lübeck, Germany; 3 Institute for Pathology, University Hospital Carl Gustav Carus, Technische Universität Dresden, Dresden, Germany; 4 Maldi Imaging Unit, Nationales Centrum für Tumorerkrankungen, partner site Dresden, Dresden, Germany; 5 Department of Visceral, Thoracic and Vascular Surgery, University Hospital Carl Gustav Carus, Technische Universität Dresden, Dresden, Germany; 6 Tumor and Normal Tissue Bank of the UCC/NCT site Dresden, University Hospital Carl Gustav Carus, Technische Universität Dresden, Dresden, Germany; 7 Fraunhofer Institute for Digital Medicine MEVIS, Lübeck, Germany

**Keywords:** Biomarkers, Cancer, Computational biology and bioinformatics, Oncology

## Abstract

Rare tumor diseases are difficult to diagnose and there is a lack of routine diagnostic procedures. Approaches must be found that allow comprehensive identification and evaluation of prognostic relevant target proteins or transcripts. Analyzing rare ampullary cancer, respectively, to their prognosis and predictive factors by machine learning (ML) based matrix-assisted laser desorption/ionization (MALDI) time-of-flight (TOF) imaging is a first step towards providing new solutions for diagnostics of those cancer samples. In this study, we investigated a cohort of ampullary adenocarcinomas, including intestinal, pancreatic and cases of unknown subtypes, to identify differences in the proteome. Human formalin-fixed paraffin-embedded (FFPE) tissues were pathologically assessed, immunohistological stained, MALDI Imaging detected, and ML-related analyzed. We enable MALDI imaging as a diagnostic complement for immunohistochemical analysis and provide a MALDI Imaging neural network for broad application in tumor diagnostics. Moreover, using tools from ML model explainability, we determined a small subset of influential *m/z*-values from the trained models. The transformation of locally established ML networks dependent on one proteomic application source to other similar application sources (without peak picking or other pre-processing) is the basis for future rare cancer patient data collection.

## Introduction

Ampullary cancer (carcinoma of the papilla of Vater) is a rare malignant disease of the gastrointestinal tract (GI)^1,2^. Compared to other gastrointestinal tumors, such as pancreatic cancer, this is associated with a slightly better life expectancy, likely due to its localization and earlier symptoms (45% with 5 year survival rate)^3–5^. Three main ampullary carcinoma subtypes are described based on histopathologic features: intestinal, pancreatobiliary, and mixed type. When compared by subtype, they differ in prognosis, 20% for the pancreaticobiliary subtype to 88% for the intestinal subtype (5 year survival rate)^4^.

Histological classification of these cancer subtypes mainly distinguishes between two molecular phenotypes, the intestinal and the severe pancreatobiliary type^6^. However, prognostic markers or molecular details are missing in clinical practice; instead, reliance is placed on known intestinal or pancreatic (cancer) cell markers. Additionally, there is a lack of molecular breakdowns to understand the different cellular development and behavior under therapeutic conditions. Currently, the tumor markers CDX2, CK7, CK20, and Muc1 in particular are used in the identification of subtypes. Histologic subtype and CDX2 and MUC1 expression were significant prognostic variables. Patients with a histomolecular pancreaticobiliary phenotype (CDX2 negative, MUC1 positive) revealed poor prognostic values^6,7^. However, these markers repeatedly show ambiguous expression patterns and do not show any specification of the mixed type, a third histologic undifferentiated type^8^. It is obvious that new options for classification must be explored here.

A clinically novel approach for the diagnosis of spatial tumor protein distribution is called MALDI imaging. It combines two main areas of bioanalytical standards: (1) the detection and analysis of proteins by time-of-flight (TOF) mass spectra and (2) the histology of tissue structure. With this method, regions of interest (ROI) defined by pathological evaluation can be translated into mass spectra and used for subsequent bio-statistical analyses of protein occurrence. In addition to the findings from different spectra patterns, e.g., in tumor versus non-cancerous tissue areas, morphologically and immunohistological indistinguishable tumors can be examined for proteome-dependent heterogeneity and subdivided into new cancer subtypes, e.g., pancreatic cancers. In addition to the specificity of the data patterns depending on the tissue type, MALDI imaging also shows a high relevance for use in the initial identification of the origin tissue. For example, to identify cancers of unknown primary, quantifying heterogeneity or the discovery of possible new biomarkers or therapeutic targets^9,10^. Tissues altered by therapies such as radiation show equally distinct characteristics and allow, among other things, therapy responses to be visualized. However, analyzing the large datasets produced by omic approaches as well as comparing data from different labs poses a problem.

The most established methodology for automated analysis of MALDI spectra relies on univariate analysis based on finding single discriminative *m/z* values^11,12^. While successful, such an approach is not suitable when the discriminative features become more complex. For these cases, researchers have previously employed classical machine learning tools such as decision trees^13^, linear classifiers^14^, support vector machines^15^, and more^16–19^. These are often utilized in combination with a dimensionality reduction method such as peak picking, principal component analysis (PCA)^20^, or other approaches^16,17,21^. In recent years, neural networks have achieved great success in machine learning tasks within image processing, natural language processing, and are gaining adoption in further fields as well, including MALDI mass spectrometry imaging (MALDI MSI)^17,22–25^. Neural networks have proven themselves to be powerful discriminators by being highly flexible and only relying on learned features. The last part is especially relevant for processing MALDI spectra, as it foregoes the need for dimensionality reduction.

In this work, we take steps towards improving cancer classification using neural networks with a focus on clinical usability. As a first application of this approach, we study the discrimination of cancerous and non-cancerous tissue regions directly from their measured MALDI spectra. To highlight the clinical usability of the results, we use model interpretability methods to highlight what combinations of *m*/*z* values were important for the neural network’s decisions. This allows our method to plug into existing MSI workflows such as peptide identification for biomarker discovery. Finally, apply our method to whole slide segmentation, showing how our method scales to real-world sample sizes.

## Results

### Classification results

Classification results are generated in different steps using three different methods: ROC, logistic regression and deep learning. Due to the size of the dataset, we evaluate each method using 3-fold cross-validation. We use the same splits for all methods.

### ROC analysis

As a basic approach, we perform ROC analysis inspired by Klein et al.^11^ to determine discriminative *m/z* values between cancerous and healthy tissue. For each cross-validation fold, we execute the following pipeline:

1. We compute the area under the receiver operator characteristic (AUROC) for each *m/z* value given the spectra in the training set.
2. We pick the three highest-scoring *m/z* values and use them as candidates for a univariate threshold-based classification model.
3. For each candidate *m/z* we search for the threshold which maximizes the balanced accuracy (b.acc.) as measured on the validation spectra.
4. We pick the *m/z* value and threshold which scores the highest b.acc. and apply it to the test spectra.

**Fig. 1:**
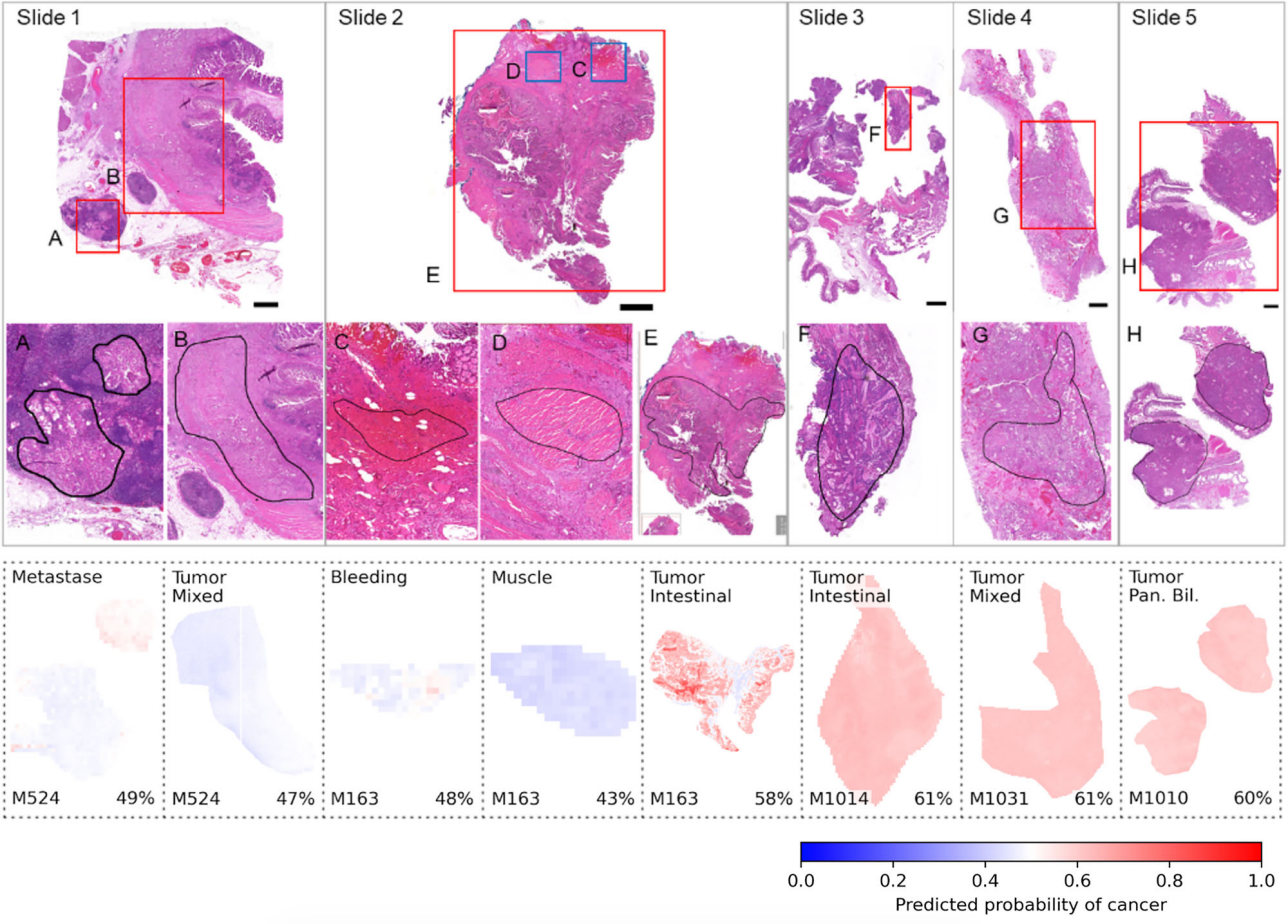
Prediction results from the test set of each cross-validation fold. Slide 1: mixed type of ampullary cancer including lymph node metastasis and primary tumor. Slide 2: intestinal type of ampullary cancer. Slide 3: intestinal type of ampullary cancer, less tumor content. Slide 4: mixed type of ampullary cancer. Slide 5: pancreatobiliary type of ampullary cancer with atypical morphological appearance. Top row: Microscopy image of full H&E stained tissue section. Regions of interest are marked with red boxes. Middle row: Zoom-ins on annotated tissue regions of interest from the top row. The black outlines indicate tissue regions annotated by the trained pathologist as either only cancerous tissue or only non-cancerous tissue. Letters A-H show the correspondence with marked regions in the top row. Bottom row: Predictions results for each measurement site of the MALDI-MS images. Each region corresponds to the annotated region marked in the H&E image from the middle row. Blue pixels represent a prediction for non-cancerous tissue and red for tumor (cancerous) tissue. The brightness of the color represents the certainty of the model. Each image shows the tissue type as marked by the pathologist (top left), patient nr. (bottom left) and predicted the probability of cancer for the whole tissue region (bottom right). Scale bars define 2 mm.

For each cross-validation fold, we evaluate the performance of the model on the test set in two ways. First, we compute balanced accuracy over all individual spectra. Second, we compute a per annotated tissue region score; we sum the predicted class probabilities for all spectra belonging to a tissue region and then pick the highest scoring class as the single prediction for the tissue region. Note that a tissue *region* refers to an annotated subsection of a full tissue *section*, see Fig. 1. This second evaluation is useful as it leverages the spatial component of MALDI MSI data to produce more robust predictions. The result of the analysis can be seen in Table 1. Only fold II shows promising results, while fold III shows worse than random performance.

### Logistic regression

As an additional basic method, we evaluate a logistic regression model, since such models have previously been used to classify MALDI spectra^25^. The logistic regression model makes a decision based on the whole spectra. We use the implementation from scikit-learn^26^ and use L1 regularization with strength 1.6 × 10^4^ and “balanced” class weights (samples are weighted with inverse proportion to class frequency). We select the regularization strength that gives the best average performance on the test sets for a best-case scenario for logistic regression. The rest of the evaluation is the same as for the univariate ROC-based model with the exact same cross-validation splits. The results are summarized in Table 1. For folds I and III, logistic regression provides a notable improvement. For fold II, it has the same performance for the tissue regions but is worse when evaluated spectra-wise.

**Table 1:** Achieved balanced accuracy (mean of sensitivity and specificity) on the test splits for each cross-validation fold for each tested model

	B.acc. spectra	B.acc region						
Method	Fold I	Fold II	Fold III	Mean	Fold I	Fold II	Fold III	Mean
ROC	0.67	0.73	0.47	0.62	0.58	0.75	0.43	0.59
Log. Reg.	0.95	0.60	0.65	0.73	1.00	0.75	0.67	0.81
NN	0.84	0.78	0.60	**0.74**	0.92	0.93	0.83	**0.89**

The first four columns show the balanced accuracy evaluated over individual spectra. The last four columns show the results when predictions are averaged over each tissue region. The best mean metrics have been highlighted with bold.

### Improving results with neural networks

We evaluate the neural network (NN) model in the same way as the ROC analysis and logistics regression models using the same cross-validation splits. The balanced accuracy results are summarized in Table 1.

Compared to the results of the classical approaches, the NN model provides an appreciable performance increase when averaging predictions over the tissue regions.

The spatial layouts of the prediction probabilities are shown in Fig. 1 for fold I. Predictions for folds II and III can be found in Supplementary Fig. 1. In fold I, the model mispredicts the metastasis and mixed type tumor for patient M524. In fold II, the model only misclassifies the pan. bil. tumor tissue from patient M144. In fold III, the model misclassifies the tumor tissue from patients M149, M150, and M523. Furthermore, the predictions show a larger degree of false positives and negatives which is also reflected in the low balanced accuracy—both for the baseline and the NN model. We note that for all models, the misclassifications at the tissue region level are all false negatives—tumor tissue is misclassified as non-concarous tissue. Finally, all models show a bias towards low confidence predictions, i.e., they tend to predict probabilities that are close to 0.5.

### Model explainability

To further understand why the trained models make their decisions, we compute importance scores for each *m/z* value using the integrated gradients (IG) method by Sundararajan et al.^27^. Given an input spectrum and target class, IG assigns an importance score to each *m/z* value that specifies whether the intensity of this *m/z* value contributes positively or negatively towards predicting the target class. We set the target class to cancerous tissue, meaning positive scores count towards classifying as cancerous and negative scores count towards classifying as non-cancerous tissue.

For the attribution scores, we have two desirable properties. First, we want the scores to be *sparse*, i.e., only a few *m/z* values should have scores with large-magnitude scores. Sparse scores are necessary for human interpretability and current biomarker workflows based on, e.g., peptide identification. Finally, non-sparse scores indicate that the model is basing its decisions on random patterns in the training data instead of real patterns. Second, we want the scores to be *consistent*, meaning that (1) the same sparse subset of *m/z* values should be chosen for each sample and (2) *m/z* values with, e.g., positive scores for one sample should also be positive for other samples. If scores for an *m/z* vary between negative and positive between samples, it is less reliable as a biomarker. Even worse, if the scores for different samples *within* each cross-validation fold are consistent but vary *between different* cross-validation folds, this indicates overfitting to a spurious correlation for that *m/z* value.

**Fig. 2:**
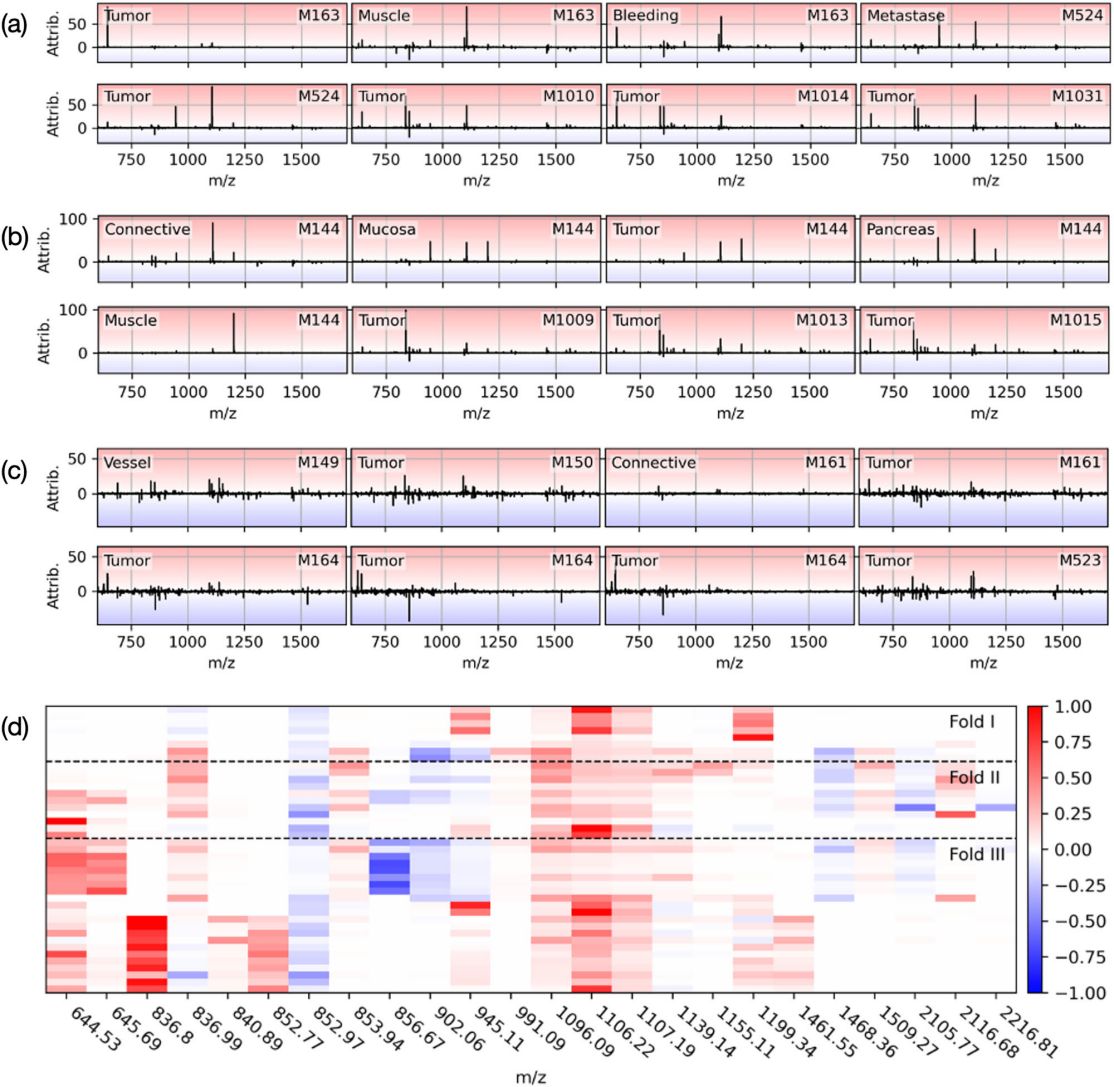
Attribution scores for test tissue samples. The graphs in (**a**–**c**) show how each *m/z* value contributes towards the model decision for representative tissue samples (full results in Supplementary Fig. 3): positive scores mean this *m/z* value generally contributes towards classifying as cancerous tissue, negative scores contribute towards classifying as non-cancerous and close to zero mean this *m/z* value rarely influenced the decision. Only scores from *m/z* 600 to 1700 are shown to improve visual clarity. **d** Summarizes the most important scores across all samples. **a** Attribution scores for representative samples in cross-validation fold I. **b** Attribution scores for representative samples in cross-validation fold II. **c** Attribution scores for representative samples in cross-validation fold III. **d** Attribution scores for most important *m/z* values across all samples. Rows correspond to tissue samples, columns to important *m/z* values and pixels show the attribution score. Scores for each cross-validation fold have been normalized w.r.t. the maximum score magnitude in that fold.

We first investigate sparseness. Figure 2a–c shows the mean importance score for each tissue sample. The mean is computed over all spectra for the sample. Additionally, we divide the mean attribution scores with the standard deviation of the scores for that tissue sample, in order to make scores across tissue samples comparable.

While the NN model has access to all *m/z* values of the spectrum for classification, it is clear that it has learned to focus on a sparse subset of *m/z* values, as desired. Furthermore, most of the attribution weight is towards classifying as non-cancerous. To check for consistency, we summarize the scores for the most important *m/z* values in Fig. 2d. Specifically, we find the *m/z* values where the absolute value of the attribution score is at least 20% of the maximum score for at least one tissue sample. The figure shows how the models from each cross-validation fold have learned to focus on a mostly consistent set of *m/z* values—especially the models in fold I and II, which were also the best-performing models. Even though the training set is different for each cross-validation fold, the same *m/z* values have high magnitude attribution scores although the exact weights differ from sample to sample. The sign of the *m/z* values with high attribution scores is also consistent across models, except for a few *m/z* values, e.g., *m/z* 836.99 and *m/z* 945.11.

**Fig. 3:**
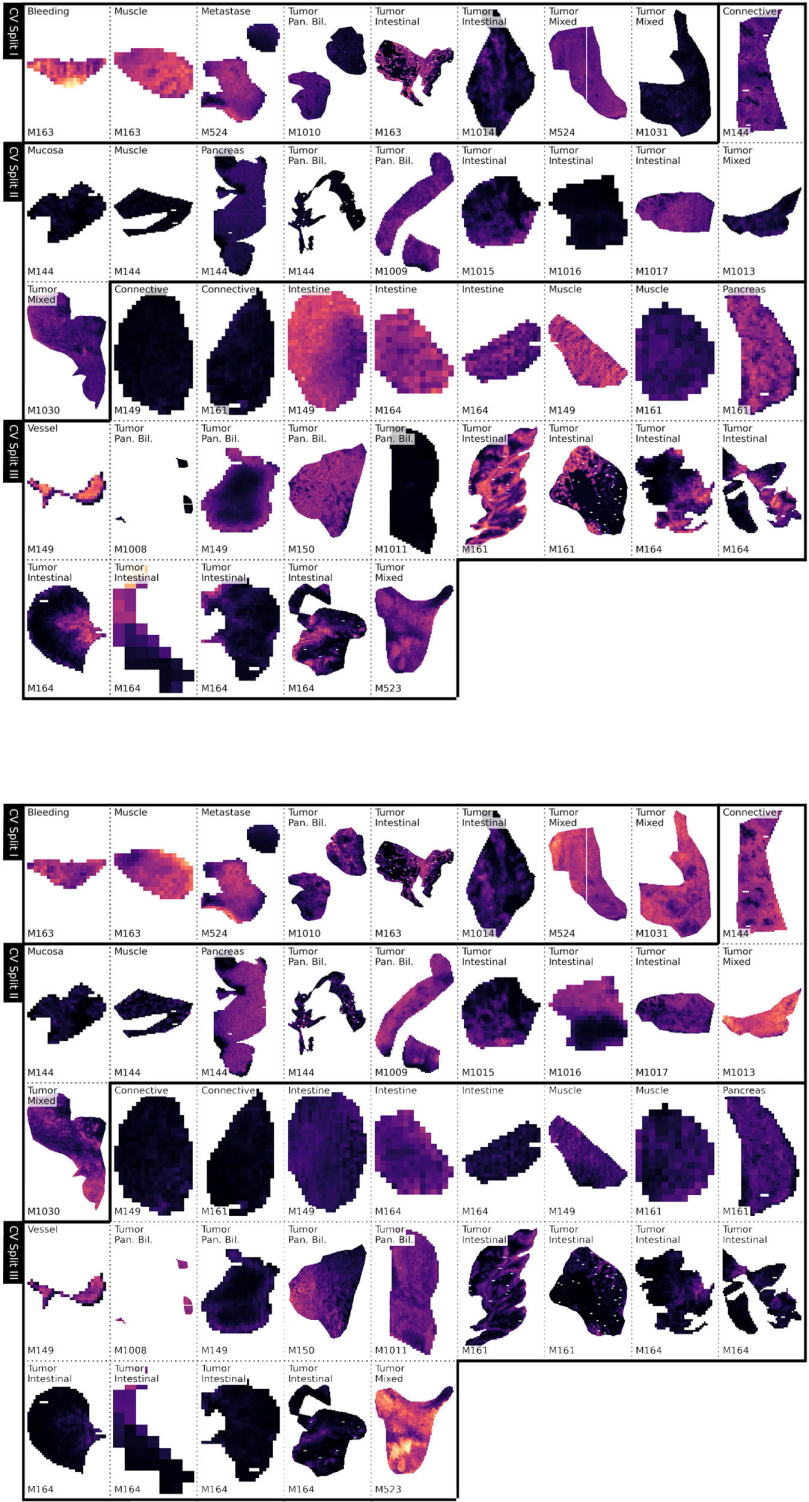
Intensities at most discriminative *m/z* values (*m/z* 852.97 and *m/z* 1106.22) shown for the test tissue samples in each cross-validation. Top: Intensities at *m/z* 852.97. Bottom: Intensities at *m/z* 1106.22.

We visualize the intensities of the original MSI data at *m/z* 852.97 and *m/z* 1106.22 in Fig. 3. These *m/z* values are chosen based on Fig. 2d as they have the highest and most consistent attribution scores over the three cross-validation folds. According to Fig. 2d, *m/z* 852.97 should generally be associated with non-cancerous tissue. For cross-validation split I and III, this is also the case with several non-cancerous tissue samples showing high intensities. Similarly, *m/z* 1106.22 should be associated with cancerous tissue and several cancerous tissue samples also show elevated intensities at this *m/z*. However, common for both *m/z* values is that they cannot serve as robust classifiers on their own. Despite generally indicating non-cancerous tissue, there are samples for *m/z* 852.97 which are cancerous but show high intensity values and vice versa for *m/z* 1106.22. Furthermore, we show in the supplementary materials (see Supplementary Table 5) that using the *m/z* values from Fig. 2d as the features for a logistic regression model still performs worse than the NN model. Therefore, while Fig. 2 gives insight into important *m/z* values it is the ability of the NN model to *non-linearly* combine them which provides it with its classification power.

### Segmentation of full tissue sections

**Fig. 4:**
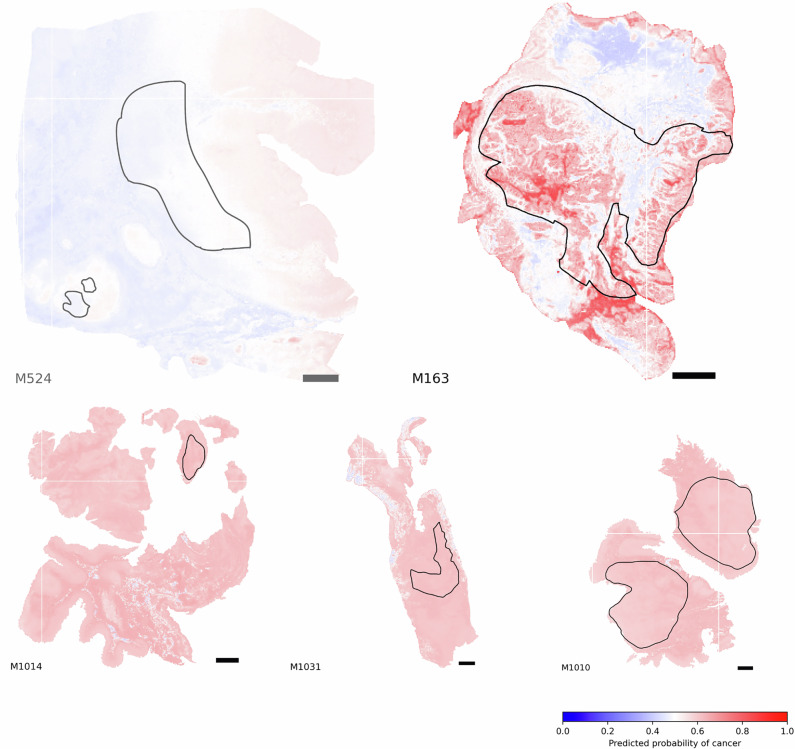
Results of applying the model from cross-validation split I to full tissue sections MALDI-MS images. Blue pixels correspond to regions the model classifies as non-cancerous, red regions as cancerous and white regions as unsure. Black outlines delineate tumor regions from Fig. 1. Regions predicted as cancerous correlate well with the annotated regions in Fig. 1. Scale bars define 2 mm.

We apply the model to the set of MALDI MSI data of full tissue sections to evaluate the ability of the model to distinguish cancerous regions from non-cancerous in one image. We only use the model and tissue sections from the test set of cross-validation fold I since this had the highest performance on spectra-level classification. The results can be seen in Fig. 4. The models perform inference at a speed of roughly 65,000 spectra/s. For samples M524 and M163, there is a good correlation between the regions with a high estimated probability of being cancerous and the annotations in Fig. 1.

The model correctly identifies the metastatic region in the bottom left of M524 as well as the cancerous area in the center of M163. Additionally, for sample 524, the model also correctly identifies the right part of the tissue as cancerous even though it was not part of the original annotated dataset. From Fig. 1, it is apparent that some parts of the MALDI spectra correspond to areas without tissue. The model tends to predict these regions as cancerous. For sample M1014, the model correctly classifies the top region as cancerous. However, since the histological results show fewer cancer markers in the lower region, the model should classify more as non-cancerous tissue, or, at least, cancer with less confidence than the top region. For sample M1031, it correctly marks the whole tissue as cancerous except the top left region which consists of inflamed but non-cancerous tissue. Finally, for sample M1010, the model correctly detects the top, right and low-center parts of the lower region as cancer. However, it fails to classify the remaining regions as non-cancerous tissue even though the confidence of the predictions is generally lower.

Note that these visualizations directly show the predicted probability of the tissue classes of interest. This makes them different from classical visualization of *m/z* slices based on, e.g., ROC analysis, which only shows values that are, to a greater or lesser extent, correlated with tissue classes. However, these prediction probabilities still reveal the structure of the underlying tissue, as shown in Supplementary Fig. 2.

## Discussion

Ampullary carcinomas, also known as papillary carcinomas, are malignant diseases of the gastrointestinal tract that occur at the papilla Vateri. Patients with this disease have a poor prognosis with a median overall survival of less than 30 months^28^. In addition, the tumors are located in a difficult-to-operate area between two different mucous membranes^29^, namely those of the pancreas and the intestine. This ambiguity requires a precise diagnosis in order to distinguish between the different pathological origins and different treatment characteristics. Immunohistological markers are intended to help here, but they do not provide a clear answer. Rather, it appears that the subtyping of ampullary carcinomas requires a weighing of the available markers (CDX2, CK7, CK20, muc1, etc.) until no distinction is possible and a “mixed type” is then referred to. This highlights a knowledge gap in ampullary carcinomas that diagnostics have not been able to address.

These complex tumor histologies, described even briefly by genetic alteration^8^, which do not allow a clear diagnosis, are the target group for new multiplex imaging methods. MALDI imaging takes this approach and, in addition to spatial localization, also shows the possibility of detecting previously undefined (non-targeted, label-free) proteins and distinguishing between regions of interest. This allows tumor types to be compared even without known markers, making them ideal for the challenge of diagnosing ampullary carcinomas. MALDI imaging has already demonstrated on multiple occasions that it can not only distinguish between tumor tissue and non-cancerous tissue, but also verify the origin of the tissue^22,30^. Even rare tumor diseases could be better detected with the help of imaging mass spectrometry and is therefore increasingly being discussed here as a diagnostic method to compensate for the lack of markers^31^.

The size of the generated data images is significantly larger than is usual in histology and requires automated machine learning or networks with NN in order to process time-critical analyses^22,32^.

**Fig. 5:**
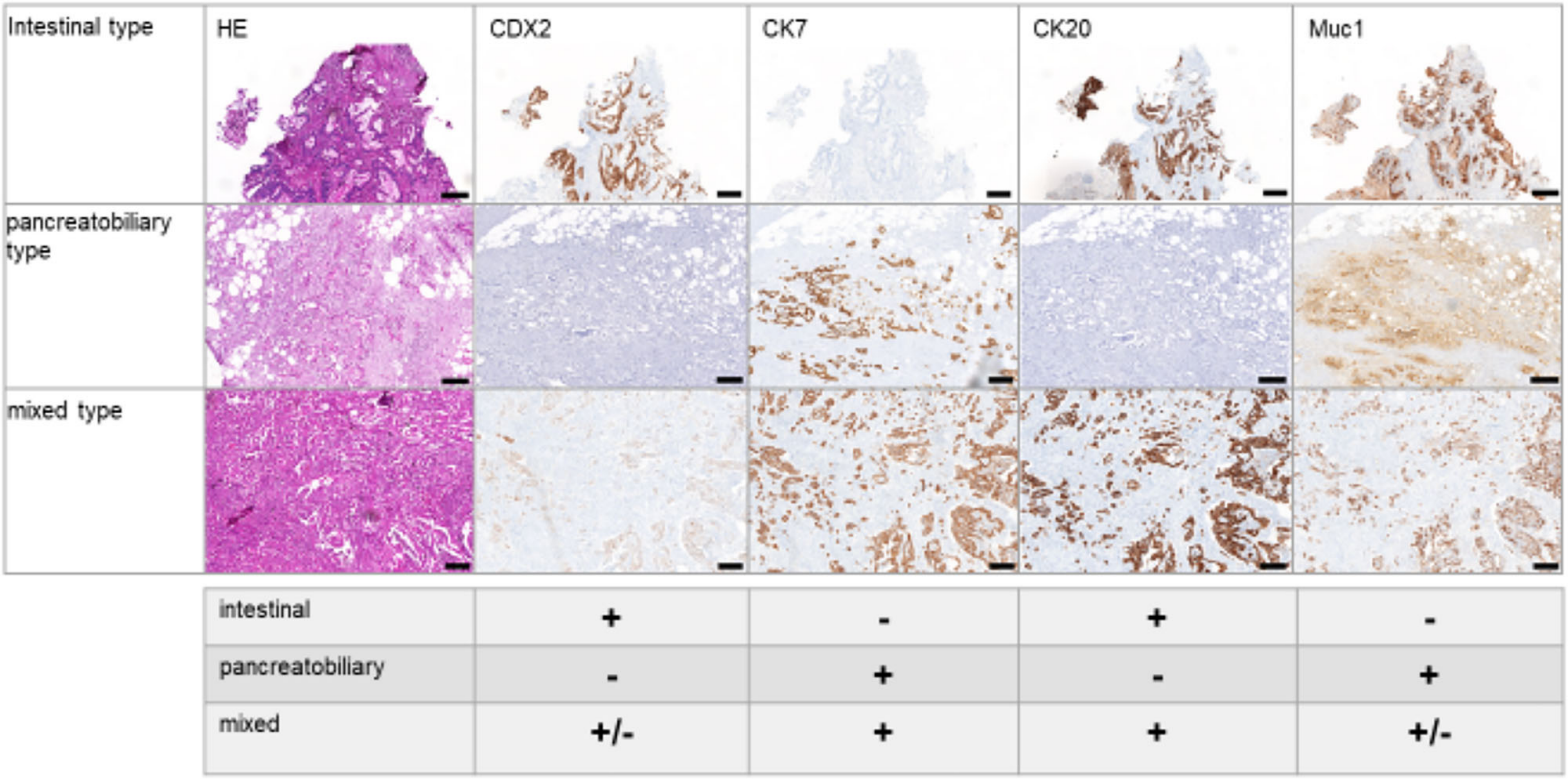
Immunohistological staining of tissue sections of ampullary carcinoma. The used markers CDX2, CK7, CK20, and Muc1 show different expression levels in the cancer subtypes (intestinal CDX2 +, CK20 +, Muc1 +; pancreatobiliary CK7 +, Muc1 +). Scale bar: 200 µm.

As shown in Table 1, the performance of the NN models is notably higher than the ROC-based approach. This demonstrates the advantage of models that can combine information from multiple *m/z* values. Furthermore, when the trained NN model is applied to whole-slide MALDI-MSI images (c.f., Fig. 5 and Supplementary Fig. 2), it shows promising abilities in highlighting cancerous and non-cancerous tissue regions. The NN models are also very efficient compared to established methods—processing over 65,000 spectra per second.

Furthermore, instead of relying on peak picking or other pre-processing steps, our NN models automatically identify important *m/z* values as part of the training process, as shown by Fig. 2. The *m/z* values selected by the NN models are also consistent across cross-validation folds. As the training data is different for each fold, this supports the conclusion that the chosen *m/z* values represent real discriminative features and are not simply a result of overfitting to specific samples. Moreover, being able to extract important *m/z* values from the models after training allows us to integrate the NN models into existing MSI workflows such as peptide identification for biomarker discovery. As the identified *m/z* values are based purely on importance scores and not the measured intensity in the spectra, this process could help with identifying biomarkers from low-concentration molecules. However, the quality of the importance scores also depend on the trained NN model. For example, the importance scores for fold III are generally more noisy (c.f. Fig. 2). This may be a result of the NN model for this fold being more uncertain as evidenced by the less homogeneous predictions on the fold III test samples, c.f. Supplementary Fig. 1. Additionally, before any clinical implementation, such *m/z* signatures should undergo further validation by full MS/MS identification to ensure they are not a result of technical artifacts. We relegate further investigations of this to future work.

One must also stress that the *m/z* signatures identified via importance scores cannot directly replace the NN model. This is immediately obvious from the fact that the exact weight of each coefficient varies from tissue sample to tissue sample. This is also shown in Supplementary Table 5: a linear classifier using the highlighted *m/z* signatures has a lower performance compared to the full NN model.

Analyzing the errors of the model, we notice that all errors are false negatives, i.e., classifying cancer tissue as non-cancerous. This indicates the model has a slight bias towards the non-cancerous tissue which—in this work—is the minority class. However, when applied to full tissue sections (c.f., Fig. 4) this trend is reversed, i.e., the errors of the NN model are mainly false positive predictions. Also noticeable is the low confidence of the predictions, as is evident in Figs. 1 and 4. A likely cause of this is the small dataset which may cause the features learned by the model to be less robust. Also, we must apply a high amount of regularization to the model to avoid overfitting. Yet, removing this regularization has large negative effects on performance as shown in Supplementary Table 4.

It also remains unclear what form of quality standards, e.g., negative controls, can be used for comparative analysis of such heterogeneous tumor structures. When referring to very conservative structures such as muscle or blood, it is easier to differentiate them from tumors than when comparing the original tissue and the tumor itself, often because they are located close to each other spatially and paracrine modifications cannot be ruled out.

Naively, this problem can be solved by creating a larger dataset. However, we see a clear tendency that the NN model produces higher quality predictions for the newer samples with M-number over 1000. These samples generally have more confident and less noisy predictions, c.f., Fig. 1. This is encouraging as it indicates a positive development in measuring quality. However, it also means that great care must be taken during data capture with the aim of growing existing datasets. Due to the high time and resource requirements involved in collecting MALDI MSI data, significant increases to dataset sizes will require merging datasets from longer time periods and/or different labs. However, as shown in Supplementary Table 4, differences in the spectra alignment or intensity distributions between tissue samples can cause a marked performance degradation. Therefore, in our view, developing more effective ways of standardizing MALDI MSI data from different machines is of critical importance. This is what will enable the creation of large datasets to unlock the full potential of NN-based analysis approaches.

The origin of ampullary tumors remains unclear to the present day. Carcinogenesis, particularly with regard to the different morphologies of intestinal, pancreatobiliary, or even mixed types, appears to be poorly understood. Genetic analyses of a panel of 24 gene signatures were also unable to assign mixed types to the other entities^8^. This suggests that mimicry or the favoring of one tumor has an effect on the other epithelium.

The combination of these new approaches of MALDI Imaging and ML demonstrates the potential of these analytical methods. In the future, diagnostic monochromes will be replaced by high-multiplex methods, which, in addition to analyzing known target proteins, also detect hidden proteins. Clinically, this is crucial in order to be able to answer future questions from this source. Similar to a genome database, these datasets will use ML algorithms to map the heterogeneity, but also the similarities, of tumor entities. Physicians themselves will then be able to use the new representations for diagnosis and gain insights from the multifactorial analysis. In this way, cluster maps of patient tissue can contribute to the development of new therapies, and new forms of multiplex analysis can be used to identify findings in pathology. A shift in thinking from classic diagnosis to the recording of complex relationships will bring clinical care closer to research. And this ultimately benefits patients, who will have access to the latest therapies.

In conclusion, we demonstrated that a deep learning approach can produce an effective model for detecting cancerous tissue based on spectra from MALDI-MSI data. This was illustrated on a challenging clinical dataset containing multiple tissue types and cancer subtypes. When compared to classical approaches such as ROC-based classification or logistic regression, the NN model outperformed both on classification performance. Furthermore, it achieved this without dimensionality reduction techniques such as peak-picking or PCA; instead, the model used the full spectrum as its input which minimizes the need for expert data pre-processing. Once trained, we demonstrated that the NN model can be deployed to detect cancerous regions in full tissue slides containing hundreds of thousands of spectra. This illustrates the potential for deploying such NN models as bulk detection systems in clinical practice.

Moreover, we show how model explainability tools can be used to extract important *m/z* values from the trained NN model. With this, we verified that, through our training pipeline, the models learn to focus on a small and consistent set of *m/z* values. This also allows the NN models to be used as part of existing clinical workflows based on, e.g., peptide identification for biomarker discovery. In essence, extracting important *m/z* values from the trained model can be used as a replacement for peak picking. Different from existing approaches, these *m/z* values are based on learned features that discriminate between the classes of interest and not the intensities in the raw spectra.

To further improve the results and usability of this work, we see the following two directions as the most promising. First, the dataset needs to grow further to produce more robust models. Long term, this will necessitate merging datasets from different clinics, which means a common data standardization protocol for MALDI-MSI data must be established. This will also be necessary to make such models usable beyond the facility where they are trained. Second, while this work focused on distinguishing cancerous tissue from non-cancerous tissue, future work should focus on distinguishing between different cancer subtypes, e.g., pancreatobiliary, intestinal or mixed type. This is where current methods struggle the most to make reliable predictions and therefore where NN has the biggest potential to improve diagnostic workflows.

## Methods

### Patient sample collection

A total of 19 patients with complete immunohistochemical subtype analysis were included in the study. Tumor stage was defined according to the TNM classification published by UICC (Union of International Cancer Control) in 2010.15 In accordance with the guidelines for research on human subjects, all patients gave their informed consent prior to resection and approval was obtained from the local ethics committee at the TU Dresden (EK 59032007). By immunohistochemical analysis of the surgical specimen we identified the pancreatobiliary subtype in 32% of patients, the intestinal subtype in 42% of patients, and the mixed subtype in 26% of patients. Supplementary Table 1 shows the diverse clinical and pathological characteristics according to the morphological subtypes of the patients with ampullary carcinoma.

### Pathology assessment and annotation

All available slides of patients included in this study were re-evaluated by two pathologists. Tumors were classified as intestinal or pancreaticobiliary subtypes according to histomorphology and immunohistochemical expression profile using Hematoxylin & Eosin (HE) staining and immunohistochemical expression analyses of CK7, CK20, CDX2, and MUC1, according to World Health Organization (WHO)-criteria, see Fig. 5. Tumors with indistinct morphological and/or immunohistochemical characteristics were classified as mixed type.

### Embedding and sample fixation

The cancer sample sections were fixed in 4% formalin and transferred to embedding cassettes. The blocks were automatically dehydrated and embedded in paraffin using the tissue infiltration machine (STP 420ES, Thermo Fisher Scientific Inc., Waltham). Formalin-fixed paraffin-embedded (FFPE) samples have been stored at room temperature.

### HE and immunohistochemical staining

HE staining was performed on 1–2-µm-thick thin sections in an automated manner (Dako Omnis, Agilent Technologies, Santa Clara).

Immunohistochemical staining of CDX2 (CellMarque, diluted 1:200), CK7 (CellMarque, diluted 1:250), CK20 (Agilent/Dako, diluted 1:200, LabVision), and Muc1 (CellMarque, diluted 1:100) have been established by diagnostics standards for 1–2 µm human FFPE tissue sections. The antibodies of CDX2, CK7, and CK20 have been processed by using OptiView Amplification™ DAB IHC Detection Kit (Ventana® Medical Systems, Roche) and the automated system Bench Mark Ultra IHC Instrument (Ventana® Medical System, Roche). Muc1 antibodies have been visualized using Ultra View Amplification™ DAB IHC Detection Kit and BenchMark Ultra IHC Instrument (Ventana® Medical System, Roche). All kits have been used as manufacturer’s protocol which are documented in data sheets P/N 760-099 and P/N 860-099.

All staining methods have been done with the help of the Institute of Pathology of the University Hospital Carl Gustav Carus, Dresden, Germany.

### Sample preparation and rehydration

Indium tin oxide coated glass slides (ITO) were covered with Nonident P‑40 (1:1000) and Poly-L-Lysine in water (1:1). FFPE tumor specimens were cut to 1–2 µm sections and deparaffinized following twice xylene, isopropanol, 100% ethanol, 96% ethanol, 70% ethanol and water for 5 min each.

### MALDI imaging sample preparation

Representative tumor slices were analyzed by MALDI MSI. To unmask the binding sites, slides were incubated at 110 °C, 6 bar for 20 min in HPLC water (Zytomed). ITO slides were dried in the vacuum device for 30 min. 16 cross layers of sequencing grade porcine trypsin with 20 mM ammonium bicarbonate were sprayed on the tissue section with 10 psi nitrogen gas, 0.015 ml/min and 30 °C by HTX sprayer (HTX Technologies) and enzyme digestion followed inside a wet chamber with 50 °C for 2 h. 200 mg HCCA (alpha cyano-hydroxycinnamic acid) were solved in 14 ml acetonitrile with 6 ml distilled water and 200 µl TFA and 4 layers of HCCA matrix were sprayed on the section by HTX sprayhood (HTX Technologies). To calibrate mass sizes an external peptide mixture (Peptide Calibration Standard II, Bruker) was added to the matrix layers.

### MALDI TOF data acquisition

Samples were measured by RapiFlex Tissuetyper (Bruker) for 600–3200 *m/z* with positive ion mode and reflector detector with 1.25 Gs/s digitizer detection rate. A laser spot of five times 11 µm × 11 µm resulting in pixel sizes of 50 × 50 µm was set with 500 laser shots on a frequency of 5000 Hz for application. For spatial localization of the measurement, MALDI glass slides were scanned in before trypsin digestion and matrix covering. The digital slides were overlaid with the instrument spatial settings by using FlexImaging software (Bruker Daltonik GmbH). MALDI imaging runs were performed by using FlexControl software version 4.2 (Bruker) settings, standardized as described for all samples. Mass calibration was performed using an extended quadratic analysis method with the Peptide Calibration Standard II (Bruker).

### MALDI MSI data pre-processing

Prior to analysis, a trained pathologist manually marked 41 tissue regions in 19 patients. A total of 235,587 spectra were annotated—per patient numbers available in Supplementary Table 2. Of these regions, 15 contain only non-cancerous tissue and 26 contain only cancerous tissue. Before data export, all spectra were normalized according to total ion count (TIC) and subjected to baseline correction. However, the annotated tissue regions still have different intensity distributions. Therefore, we perform additional normalization by standardizing the spectra from each tissue area with the mean and standard deviation over the tissue area. We found this step important to the performance of our classifiers.

We evaluate all models using 3-fold cross-validation. For each cross-validation fold, there is a training, validation, and test split. The data is split such that all tissue samples belonging to a patient only occur in one split. Furthermore, the splits were stratified such that the proportion of spectra representing non-cancerous and cancerous tissue was kept approximately equal for all splits. We use the same splits for all models; detailed information on each split is available in Supplementary Table 3.

We evaluate the performance of the models using balanced accuracy (b.acc.) which is defined as the average of recall for each of the two classes. For binary classification (our case) it is equal to the mean of sensitivity and specificity. It is also equal to the area under the Receiver Operating Characteristic (ROC) curve (AUROC) calculated with binary predictions (0 or 1) instead of prediction probabilities. We choose b.acc. over normal accuracy as it is robust to class imbalance which our data exhibits. For example, for a classifier that only predicts one class, b.acc. will always be 0.5.

Finally, since the spectra from some patients were captured under different scan settings, it was necessary to register the spectra such that *m/z* values were consistent. We found this step crucial for the performance of the classification algorithms. To avoid test leakage, we conduct a separate registration for each cross-validation fold as follows: We first create a reference spectrum by averaging all training and validation (but *not* test) spectra from the 11 most recent scans. These patients are the most recently captured and were therefore already well registered with each other. Given this reference spectrum, we register the median spectrum from each tissue sample by finding the *m/z* offset that maximizes the correlation with the reference spectrum. To increase the precision of the registration, we first upsample both spectra by 4x and constrain the offset to be at most 0.8. This results in three different versions of our dataset—one for each cross-validation fold.

Afterward, all spectra were resampled such that each *m/z* bin has a width of 0.2 Da, for a total of 13,350 intensity values between 600 and 3200 *m/z*. Finally, similar to ref. ^22^, we implement a filter to discard spectra with no distinct peaks. Specifically, we compute a modified peak signal-to-noise ratio of a spectrum, in dB, given by

$$MPSNR(S)=1{0\log }_{10}(\max (S)/{{\rm{std}}}_{60}(S)),$$

where *S* is the intensities of the spectrum and std_60_(*S*) is the standard deviation of the smallest 60% of the intensities. Using only the smallest 60% of the intensities gives a more robust estimation of the noise level, as it avoids including the peaks. We discard any spectrum with an *MPSNR* of less than 12.

### Classification with deep learning

In order to automatically classify the mass spectra as belonging to tumor or non-cancerous tissue, we employ an artificial neural network. In the following, we provide an overview of neural network methods. Since we only use one neural net architecture in this work, we will limit our exposition to this and refer to ref. ^33^ for a wider survey and details. The overall goal of neural network methods is to approximate an unknown decision function from a set of examples called the training data. In our case, we wish to learn a function which takes a MALDI spectrum as the input and outputs the class of the spectrum, i.e., whether the spectrum comes from cancerous or non-cancerous tissue. The basic building block of a neural network is the “neuron”, whose output is given by a weighted sum of its inputs followed by a non-linear activation function. Neurons are then grouped into layers, where, for the first layer, the inputs are the intensity values of the spectrum. We can then add additional layers where the input to each neuron are the outputs of the preceding layer. For the final layer, we typically have one neuron for each output class followed by a function that normalizes the outputs such that we may interpret them as the predicted probability of each class.

By using many layers and neurons, neural networks are able to model highly complex decision functions by tuning the weights of each neuron. This is done by iteratively updating the weights to minimize a loss function, L, which measures how well the predictions of the network match the ground truth. If the training data is representative of the overall data distribution, the weights learned from correctly classifying the training data will then also be able to correctly classify new unseen data. However, this is rarely the case. As a result, due to the high flexibility of neural networks, they are prone to pick up on spurious patterns in the training data that, by chance, separate the classes. To mitigate this, one must apply regularization such as additional loss functions or data augmentation where several slightly modified versions of the same spectra are shown to the neural network during training.

### Training details

For our application, we train a multi-layer perceptron (MLP)^33^ with three hidden layers of size 1024, 512, and 512. We use rectified linear unit (ReLU) activations and batch normalization^34^ before each activation function. The input of the neural network is the mass spectrum and the output is the probabilities of the spectrum belonging to each of the two classes. We found it beneficial to divide each spectrum by its median intensity before passing it to the network. We train the network using the AdamW^35^ optimization algorithm with a weight decay of 1.0, β_1 = 0.9, β_2 = 0.999. The learning rate starts at 10^−6^ and is then linearly increased over the first ten epochs to 10^−4^ where it is kept constant during training. Additionally, we apply L1 regularization on the weight matrices of the model with strength 1.0 to promote sparse weights. We found this step crucial for the performance of the model.

Due to memory constraints, the optimization is performed in batches of 512 spectra at a time, split into sub-batch sizes of 128. We oversample spectra from non-cancerous tissue regions so that batches contain an equal number of spectra from cancerous and non-cancerous tissue. Finally, we utilize cross entropy as the loss function.

### Data augmentation

The performance of the trained neural network (NN) model depends on the amount and variation of the training data. Given the limited number of patients, we apply data augmentation to the training spectra to increase the size of the dataset. This aims to artificially create new data points by randomly changing the existing ones without altering their class. For example, since mass spectra inherently contain noise, adding small amounts of noise should not change their classification. By adding such transformations during training, the model must learn to ignore these variations. Instead, it must focus on consistent patterns more likely to correspond to real biological features.

Specifically, we apply the following data augmentations to the spectra:

1. *m/z*-wise dropout. Choose 5% of the *m/z* values uniformly at random and set their intensity to 0.
2. Gaussian noise with mean zero and a standard deviation of 0.02 times the maximum intensity value of the spectrum.
3. Contrast adjustment by raising all intensity values in a spectrum to a randomly chosen power in [0.8, 1.2]. Before applying the transformation, the spectrum is scaled to lie in [0, 1] and is then scaled back to the original intensity range afterward.
4. Scaling all intensity values in a spectrum with a factor chosen randomly in [0.9, 1.1].

For each cross-validation fold, we run the training for 20 epochs where one epoch constitutes a pass through all the training data. During training, we evaluate the b.acc. score on the validation split after each epoch. After training, we evaluate the highest-scoring model on the test split.

During our preliminary investigations, we also attempted more advanced models such as convolutional neural networks (CNNs)^33^, visual transformers (ViTs)^36,37^ and deeper MLPs. However, we found that these models had lower performance which we attribute to them overfitting to the limited training data.

### Model explainability via integrated gradients

We use the integrated gradients (IG)^27^ implementation from the Captum library^38^ version 0.7.0. Given a model, an input spectrum and a target class, IG integrates the gradients of the model w.r.t. the input spectrum along the path from a baseline to the input spectrum. Based on this, it computes an attribution score for each element of the input spectrum, i.e., each *m/z* value. If the score at a given *m/z* value is positive, then a high intensity at this *m/z* value contributes towards classifying the input as the target class. If the attribution score is negative, a high intensity contributes against classifying as the target class. If the score is close to 0, the intensity at this *m/z* is considered unimportant for the classification. We set the target class to cancerous tissue and use a spectrum with all zeros as the baseline input. We use the default Gauss-Legendre integration method with 1000 steps and compute global attribution, i.e., we multiply the integrated gradients with the input^39^.

## Supplementary information

Supplementary Materials

## Data Availability

The MALDI-MSI data used for training the models in this work is publicly available at 10.5281/zenodo.17140609.

## Code availability

The source code is publicly available at https://github.com/patmjen/maldi-cancer-clf.
