# Supplementary material for: Interpreting MALDI imaging data for rare types of ampullary cancer using machine learning
Source: NPJ Syst Biol Appl. 2026 May 12;12:111. doi: 10.1038/s41540-026-00705-3 (PMC13388676; doi:10.1038/s41540-026-00705-3)
Supplement: Supplementary file 1 — Supplementary Materials [file 41540_2026_705_MOESM1_ESM.pdf]

Supplementary Materials

Patient Overview

Patient	Subtype	Sex	TNM	Age
M144	pb	M	pT3, pNx, L0, V0, Pn1, G2, R1	82
M149	pb	M	pT2, pN0 (0/14 LK), L0, V0, R0 (L); G2	74
M150	mixed	M	pT2, pN0 (0/4 LK), pMx, L0, V0, R0; G2	70
M161	int	M	pT3, pN0 (0/17 LK), L0, V0, Pn0, R0 (L; CRM-); G2	64
M163	int	M	pT2, pNx, L0, V0, Pn0, G3, R1	81
M164	int	M	pT1, pN0 (0/1 LK), L0, V0, G1; R0 (L)	57
M523	mixed	F	pT3, pN0 (0/2 LK), L0, V1, Pn1, G2; R0 (L)	87
M524	mixed	F	pT4, pN1 (10/22 LK), pM1 (LYM, HEP), L1, V0, Pn0, G3, R0 (L)	72
M1008	pb	M	pT4, pN1 (1/13 LK), pMx, G3, R1	61
M1009	pb	M	pT3, pN0 (0/19 LK), L1, V0, Pn1, G2, R0 (L)	56
M1010	pb	M	pT2, pN0 (0/14 LK), L0, V0, Pn0, R0 (L); G3	70
M1011	pb	F	pT3, pN1 (5/16 LK), L1, V0, Pn0, G2, R0 (L)	84
M1013	int	F	pT2, pN0 (0/20 LK), L0, V0, Pn0, R0 (L); G.	72
M1014	int	F	pT3, pN0 (0/22 LK), L0, V0, R0 (L), G2	76
M1015	int	M	pT2, pN0 (0/14 LK), L0, V0, Pn0, G3, R0 (L)	51
M1016	int	F	pT2, pN0 (0/20 LK), pM0 (LYM), L0, V0, Pn0, G2; R0	60
M1017	int	M	pT1, pN0 (0/30 LK), L0, V0, Pn0, G2; R0 (L)	55
M1030	mixed	F	pT3a, pN1 (1/22 LK), L1, V1, Pn1, G2, R0 (L; CRM-)	50
M1031	mixed	M	pT1b, pN1(mi) (1/18 LK), L1, V0, Pn0, G2, R0 (L), CRM-	68

Subtype		Sex		TNM						Age	
int:	8	M:	12	pT1: 2	pT1b: 1	pT2: 7	pT3. 6	pT3a: 1	pT4: 2	Mean:	67.9
pb:	8	F:	7				pN0: 12	pN1: 5	pNx: 2	Median:	70.0
mixed:	5						pM0: 1	pM1: 1	pMx: 2	Std:	11.3

Supplementary Table 1: Patient samples of ampullary cancer. Abbreviations: pb = pancreatobiliary, int = intestinal, M = male, F = female, (L) = (Local).

Additional Details on Training Data

In Supplementary Table 2, we provide details on the number of annotated spectra per patient

	M144 (PB)	M149 (PB)	M150 (PB)	M161 (In)	M163 (In)	M165 (In)	M523 (Mi)	M524 (Mi)	M1008 (PB)	M1009 (PB)
Total	19212	2485	13286	29922	22883	12126	4476	16633	4007	2194
Bleed.					136					
Conn.	1699	394		427						
Intest.		654				205				
						151				
Muco.	304									
Musc.	1204	742		141	164					
Pancr.	3199			743						
Vessel		109								
Metas.								706		
Tumor	12806	586	13268	6795	22583	864	4476	15927	4007	2194
				21816		1674				
						1019				
						31				
						440				
						7742				

	M1010 (PB)	M1011 (PB)	M1013 (Mi)	M1014 (In)	M1015 (In)	M1016 (In)	M1017 (In)	M1030 (Mi)	M1031 (Mi)
Total	65998	1207	1906	2530	770	227	4684	22391	8668
Tumor	65998	1207	1906	2530	770	227	4684	22391	8668

Supplementary Table 2: Number of annotated spectra per patient and tissue region. Note that some patients have multiple tissue regions of the same tissue type.

In Supplementary Table 3, we provide the full details w.r.t. which patients are used for each cross-validation split.

Fold I			Fold II			Fold III		
Train	Val	Test	Train	Val	Test	Train	Val	Test
M149	M144	M163	M149	M150	M144	M144	M163	M149
M161	M150	M524	M161	M163	M1009	M524	M1015	M150

M523	M164	M1010	M164	M1011	M1013	M1009	M1030	M161
M1008	M1009	M1014	M523	M1014	M1015	M1010	M1031	M164
M1011	M1017	M1031	M524		M1016	M1013		M523
M1013			M1008		M1017	M1014		M1008
M1015			M1010		M1030	M1016		M1011
M1016			M1031			M1017		
M1030								

Supplementary Table 3: Patients for each cross-validation fold.

Additional NN Results

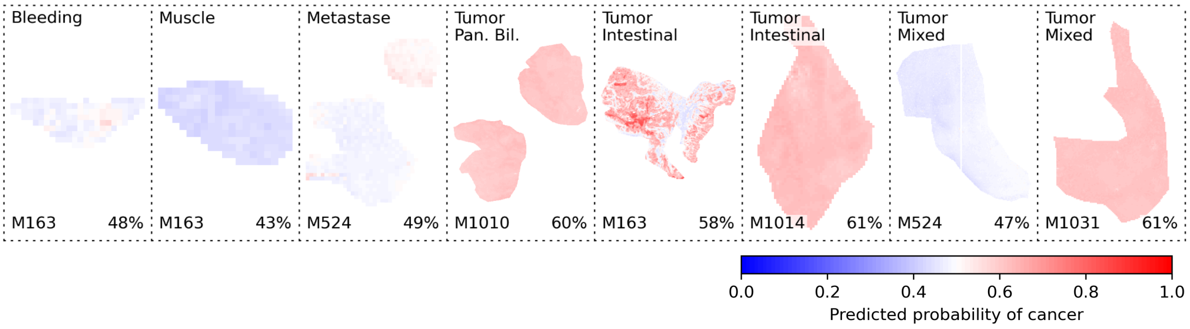

(a) Predictions for the test split in fold **I**. H&E overviews have been omitted.

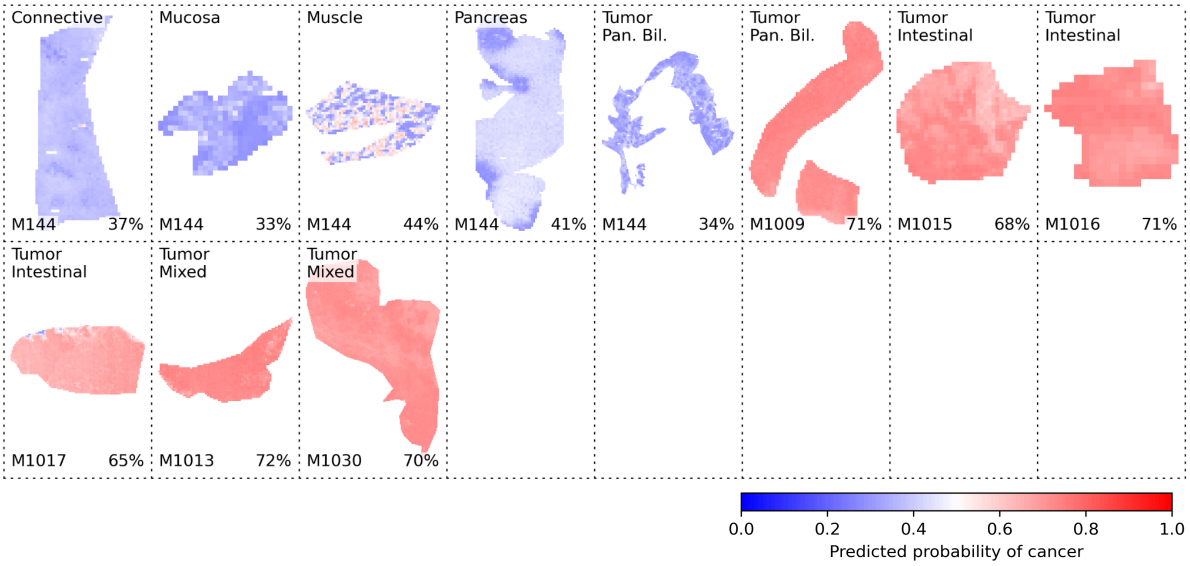

(b) Predictions for the test split in fold **II**. H&E overviews have been omitted.

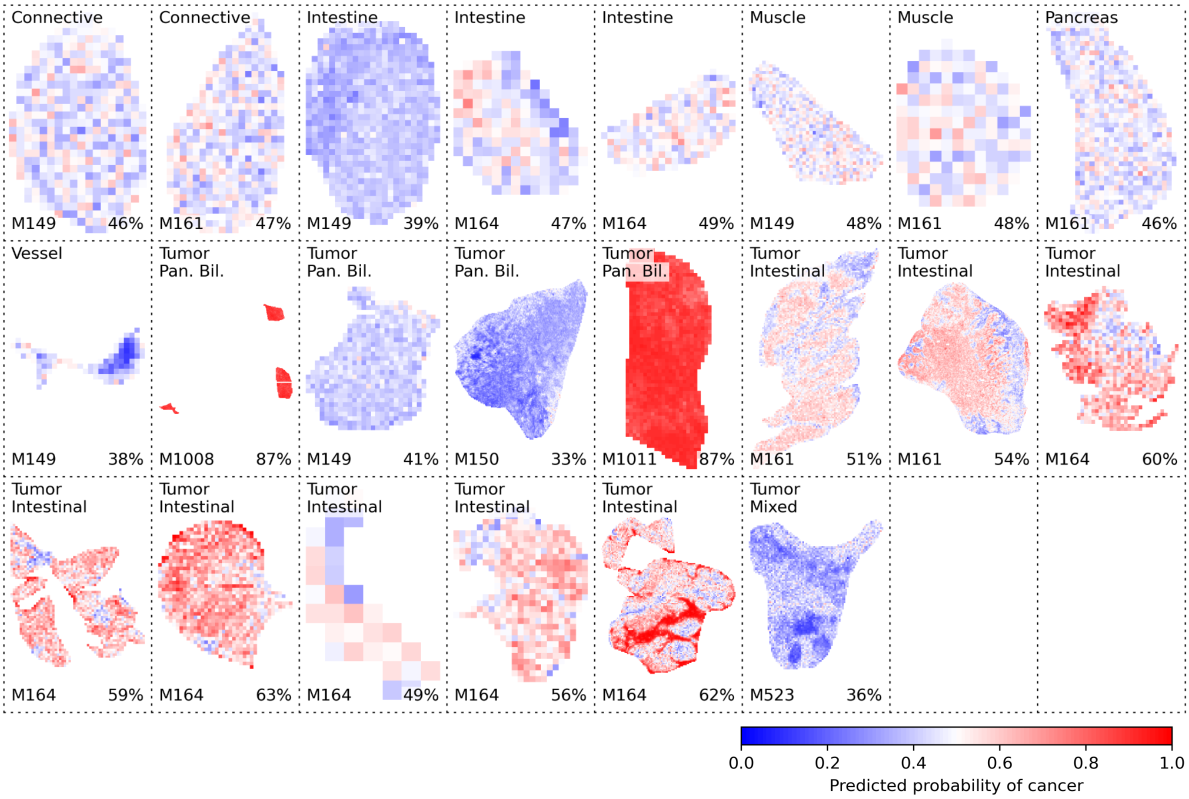

(c) Predictions for the test split in fold **III**. H&E overviews have been omitted.

Supplementary Figure 1: Prediction results from the test set of each cross-validation fold. Predictions results for each measurement site of the MALDI-MS images. Each region corresponds to the annotated region marked in the H&E image from the middle row. Blue pixels represent a prediction for non-cancerous tissue and red for tumor (cancerous) tissue. The brightness of the color represents the certainty of the model. Each image shows the tissue type as marked by the pathologist (top left), patient nr. (bottom left) and predicted the probability of cancer for the whole tissue region (bottom right).

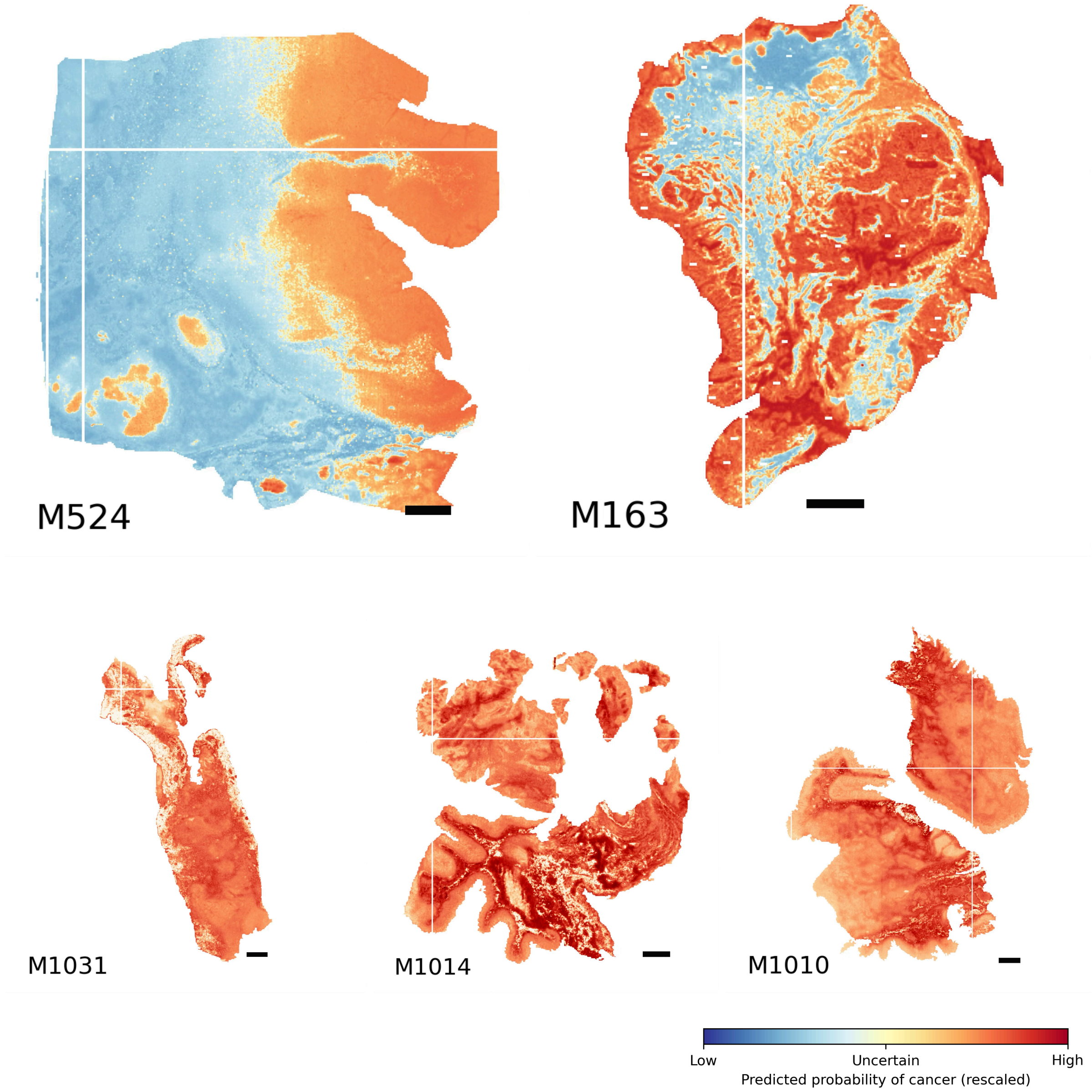

Supplementary Figure 2: Predictions from Fig. 4 shown with enhanced contrast. Blue pixels correspond to regions the model classifies as non-cancerous, red regions as cancerous and white regions as unsure. Note that the intensity range for each sample is different and chosen to maximize the contrast. Even though the coloring only shows the prediction probabilities of the model, it captures the structure of the underlying tissue. Scale bars define 2mm.

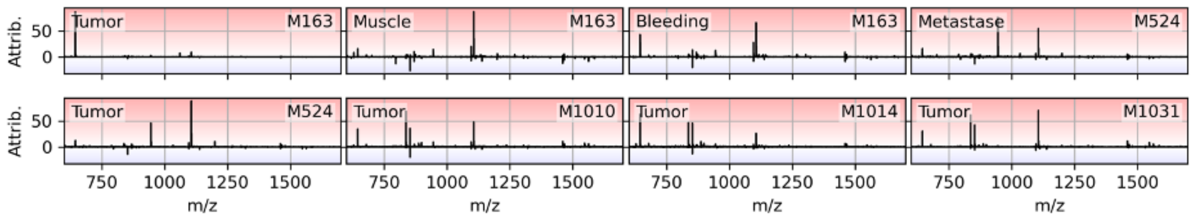

(a) Attribution scores for cross-validation fold I

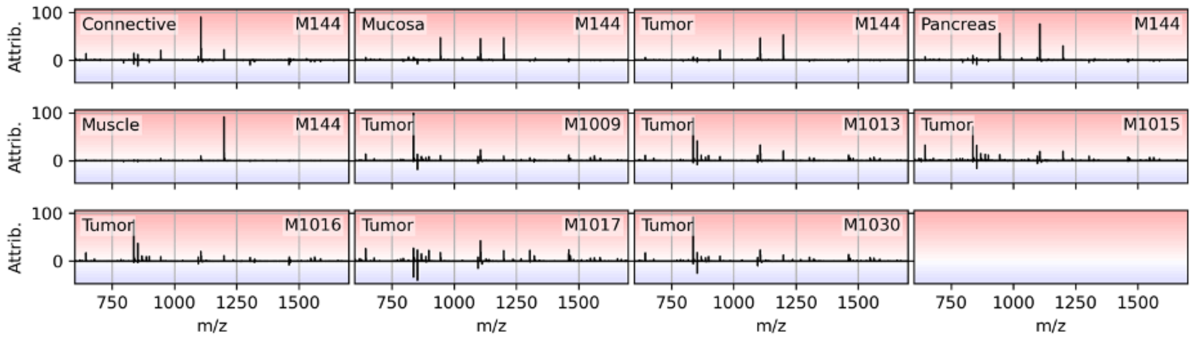

(b) Attribution scores for cross-validation fold II

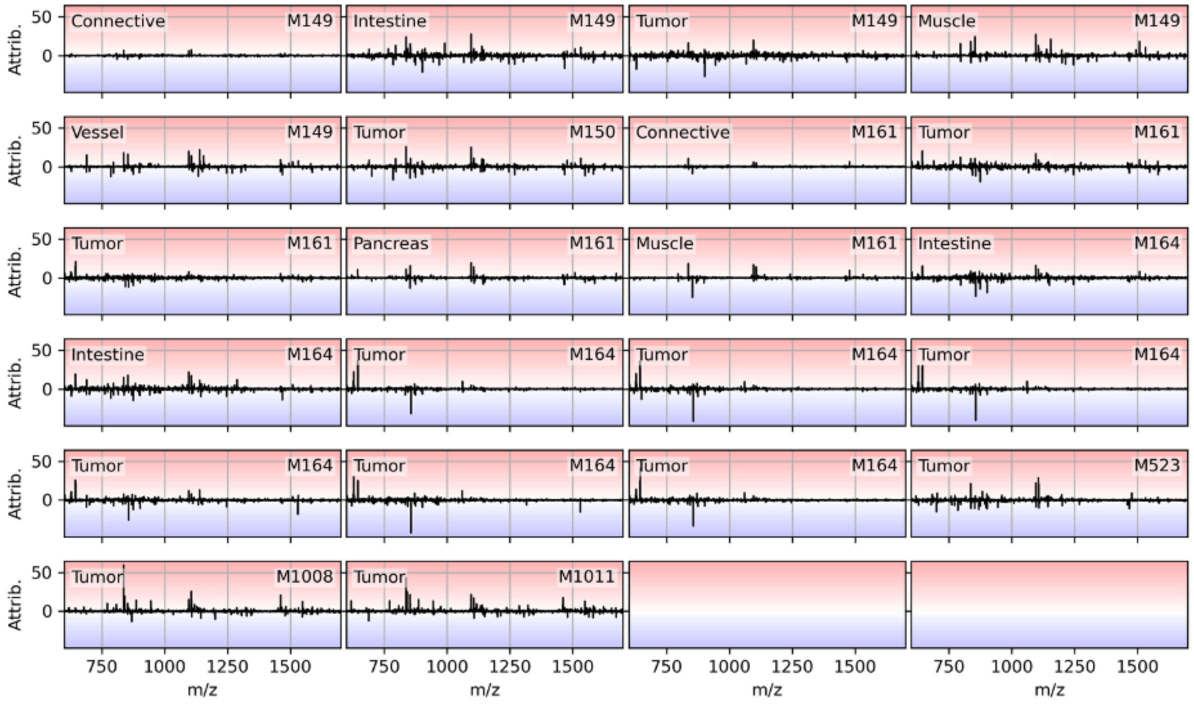

(c) Attribution scores for cross-validation fold III

Supplementary Figure 3: Mean attribution scores for test tissue samples in each cross-validation fold. The graphs show how each *m/z* value contributes towards the model decision: large positive scores mean this *m/z* value generally contributes towards classifying as cancerous tissue, large negative scores mean the *m/z* value generally contributes towards classifying as non-cancerous and close to zero mean this *m/z* value rarely influenced the decision. We only show scores from *m/z* 600 to 1700 since scores at higher *m/z* were all close to 0.

Ablation Study

To validate the benefit of each of the major components of our pre-processing and training pipeline, we perform a series of ablation experiments. Here, we remove one component from the pipeline and then re-process the data and re-train the model. Specifically, for the pre-processing we consider the tissue region normalization and the spectra registration. For the training pipeline, we consider the L1 regularization and data augmentation.

The results are shown in Supplementary Table 4. Generally, removing a component causes a noticeable reduction in classification performance. The one expectation is spectra-wise balanced accuracy, which increases once the data augmentation is removed. This, however, comes at the cost of the tissue-wise performance. We chose to prioritize the tissue-wise performance in this work.

L1 reg.	Data aug.	Registration	Normalize	B.acc spectra	B.acc. region
				<u>0.74</u>	**0.89**
Removed				0.61	0.62
	Removed			**0.79**	<u>0.82</u>
		Removed		0.66	0.67
			Removed	0.68	0.68

Supplementary Table 4: Achieved balanced accuracy (mean of sensitivity and specificity) on the test splits for each cross-validation fold using the NN model. Each row corresponds to an experiment where a component has optionally been removed. The top row is the full pipeline used in the previous sections. The best result is marked with **bold** and the second best is <u>underlined</u>.

Finally, to ensure that the improved performance of the NN model is not only due to it being able to select better *m/z* values than the logistic regression model, we train another logistic regression model using only the *m/z* values identified in Fig. 2(d). We use L2 regularization with strength 0.1 (chosen to maximize the mean tissue region b.acc) “balanced” class weights. The results are shown in Supplementary Table 5. The performance is comparable to but slightly worse than the logistic regression model trained on full spectra and considerably worse than the NN model. This shows

that the strength of the NN model lies not just in its ability to focus on relevant *m/z* values, but also its ability to non-linearly combine them.

	Fold I	Fold II	Fold III	Mean
**B.acc. spectra.**	0.55	0.61	0.75	0.64
**B.acc. region**	0.73	0.75	0.75	0.74

Supplementary Table 5: Achieved balanced accuracy on the test splits for each cross-validation fold using the logistic regression model trained on the *m/z* values identified as important by the NN model. The first row shows the balanced accuracy evaluated over individual spectra. The second row shows the results when predictions are averaged over each tissue region.
